# The Web-Based Recognise and Respond Gatekeeper Training Program: Noninferiority Randomized Trial

**DOI:** 10.2196/63204

**Published:** 2025-10-24

**Authors:** Cassandra Chakouch, Ann M Martin, Philip J Batterham, Demee Rheinberger, Fiona Shand

**Affiliations:** 1 Black Dog Institute, University of New South Wales, Hospital Road, Randwick, Sydney, 2031, Australia, 61 412454898; 2 Centre for Mental Health Research, Australian National University, Canberra, Australia

**Keywords:** suicide prevention, gatekeeper training, online intervention, noninferiority trial, suicide, mental health, depression, web-based, RCT, randomized controlled trial, Australian, Recognise and Respond, noninferiority

## Abstract

**Background:**

Gatekeeper training teaches community members to identify individuals at risk of suicide and assist them with help-seeking. A new online program, Recognise and Respond (R&R), was developed to address the need for a program specific to the Australian context, created in consultation with clinicians, researchers, and people with a lived experience of suicide.

**Objective:**

This study aimed to evaluate whether the R&R gatekeeper training is noninferior to the established Question, Persuade, Refer (QPR) online program, in improving confidence to identify and support individuals experiencing suicidal thoughts. Secondary objectives examined the short- and medium-term effects of R&R on attitudes toward suicide prevention and knowledge of appropriate responses to signs of suicide.

**Methods:**

A total of 524 participants were enrolled at baseline and were then randomized to receive access to either R&R (n=263) or QPR (n=261). The primary outcome—gatekeeper confidence in identifying and supporting an individual experiencing suicidal thoughts—was assessed using a noninferiority framework. Secondary outcomes, including attitudes toward suicide prevention and knowledge of appropriate responses to signs of suicide, were assessed using superiority testing. Outcomes were assessed via online surveys at baseline, postintervention, and the 3-month follow-up. The trial was conducted entirely online, with no face-to-face contact with participants. Data were analyzed using mixed effects linear models.

**Results:**

Participants in both groups reported significant improvements in confidence across time indicating noninferiority of the R&R program. Both groups showed significant improvements on attitudes and knowledge scores at postintervention and the 3-month follow-up, 3. to baseline. Improvement in attitude scores between baseline and postintervention and follow-up were greater for the QPR group compared to the R&R group. Gains at postintervention were maintained at follow-up for both groups considering all outcomes. There were no other between-group differences.

**Conclusions:**

The findings demonstrate that R&R is as effective as QPR online in improving gatekeeper confidence to identify and support someone having thoughts of suicide.

## Introduction

Suicide continues to be a global public health priority, claiming more than 700,000 lives worldwide each year [1]. Within Australia, over 3000 people were lost to suicide in 2022, and 1.5 million indicated that they had made a suicide plan during their lifetime [2]. A combination of systemic and personal barriers often prevents at-risk individuals from seeking help for suicidality, including limited-service availability and personal hesitation due to the perceived risk of stigma regarding mental health difficulties [3]. Two-thirds of individuals who die from suicide do not access care from a mental health professional or receive a mental health diagnosis prior to their death [4,5]. However, individuals at risk of suicide do often express their distress to those in their community, particularly family and friends, through observable and identifiable behaviors [6,7]. Effective suicide prevention strategies within the community are essential to provide support to individuals who may not seek professional clinical support but actively seek help from the community.

Gatekeeper (or community) training programs are a promising strategy to address this need and facilitate engagement with professional services for people at increased risk of suicide [8-10]. These programs provide training to members of the public or community workers who are identified as potential “gatekeepers”—individuals who may incidentally encounter people with increased suicide risk, are able to identify them by recognizing suicide risk warning signs, and who may assist the person to access care [11]. Potential gatekeepers are not restricted to roles with formal training in suicide crisis response and management. Examples of potential gatekeepers can also include primary healthcare providers, school staff, first responders, and social workers [10]. Evidence suggests that training can improve gatekeepers’ suicide knowledge, attitudes towards people with suicidal behaviors, and confidence in their ability to intervene [11-13].

The Question, Persuade, Refer (QPR) program is a well-recognized and widely used gatekeeper training program [14,15] shown to increase participants’ confidence, as well as knowledge and self-reported help-giving behaviors [16-20]. The QPR program teaches gatekeepers a three-stage model of intervention, beginning with recognizing the warning signs of increased suicide risk in an individual and questioning that person about their suicidal thoughts and feelings (Question). The program then trains gatekeepers to have supportive and reassuring conversations with individuals, during which the individual may be open to accepting referrals of help (Persuade). Finally, gatekeepers are trained to refer at-risk individuals to clinical support, intervention, and treatment (Refer). Early intervention is ideal under this model as it leads to more positive outcomes for individuals experiencing suicidality [21]. In addition to an effective face-to-face training module [22], QPR can also be delivered through an online “e-learning” module. The online module is as effective as the face-to-face format at improving outcomes on confidence for identifying and supporting those experiencing suicidality, knowledge about suicide and suicide prevention, and intentions to engage in suicide prevention [23].

**Figure 1.**
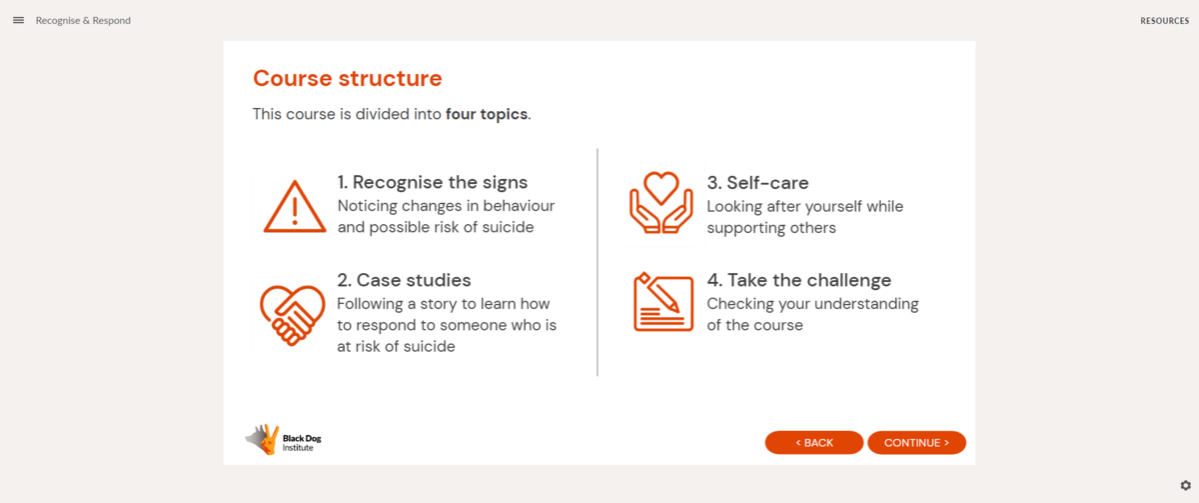
Overview of the R&R program.

The Black Dog Institute’s (BDI) LifeSpan suicide prevention trial delivered the QPR program online for community and workplace gatekeeper training within a larger trial [24]. Participant feedback indicated a need for a program with greater cultural relevance in the Australian context, rather than being culturally specific to the United States. This is consistent with literature suggesting that suicide is impacted by a complex interplay of factors including social and cultural influences [25], which may vary from nation to nation. Particularly relevant to the Australian context is the prevalence of culturally and linguistically diverse communities, for whom the role of sociocultural factors such as acculturation, stigma, social networks and family, and culturally driven intervention preferences must be considered [26]. In response, the BDI developed the Recognise and Respond (R&R) program—a gatekeeper training program that uses instructional design principles, includes diverse Australian case studies, and provides local referral options. The program was designed in consultation with clinicians, researchers, community organizations, and people with a lived experience of suicide. The R&R program consists of four topics with interactive activities, case studies depicting interactions between a person experiencing thoughts of suicide and a helper, and scenario-based challenges to test learning (Figure 1).

**Figure 2.**
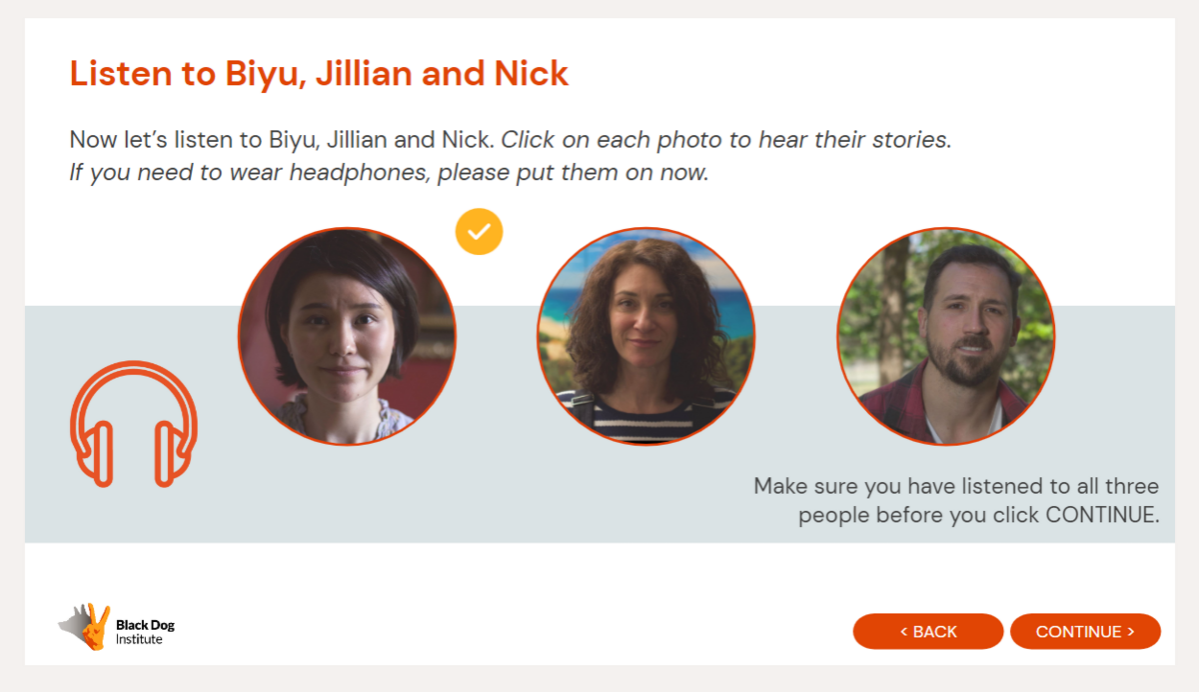
Case studies to learn how to respond to someone who is at risk of suicide.

The content covered in interactive lessons is reinforced through PDF resources that users can download and keep for future reference. The case studies included in R&R (Figure 2, images in the training program used with consent) are tailored to focus on Australian populations that are disproportionately affected by suicidal risk and behavior including Aboriginal and Torres Strait Islanders, the LGBTQIA+ (lesbian, gay, bisexual, transgender, queer/questioning, intersex, asexual) community, men, refugees and migrants, and Australians living in rural and remote communities [27]. The inclusion of these priority populations is to ensure that prospective gatekeepers are aware of the nuances that place these groups at heightened risk and to provide them with the contact details for tailored and locally relevant mental health services.

Beyond the Lifespan trial, internet-delivered gatekeeper programs have yet to be extensively evaluated in the Australian context. To leverage the potential benefits of online interventions in enhancing dissemination and accessibility [28], such as increased flexibility and cost-effectiveness [29], R&R was designed to be an internet-delivered training program. This paper reports outcomes of a noninferiority trial, a design used to determine whether a new intervention performs at least as well (noninferior) as an established intervention within a prespecified margin, rather than aiming to demonstrate superiority. This approach was chosen to evaluate whether the Australian R&R program could achieve comparable results to the established and effective international online QPR program when delivered with an Australian sample.

### Objectives of the Current Study

The primary objective of the present study is to assess whether the R&R gatekeeper training program is noninferior to QPR in improving confidence in identifying and supporting an individual experiencing thoughts of suicide at postintervention and follow-up. Secondary objectives were to evaluate the short-and medium-term impacts of the R&R program relative to QPR on attitudes towards suicide prevention and knowledge of appropriate responses to signs of suicide, using a superiority framework

It was hypothesized that participants who completed the R&R and QPR online gatekeeper programs would show a similar (noninferior) improvement from baseline to posttraining and at the 3-month follow-up on confidence in gatekeeper skills (primary hypothesis). Additionally, it was hypothesized that there would be no significant differences between R&R and QPR participants on improvements in attitudes towards suicide prevention, and knowledge of appropriate responses to signs of suicide (secondary hypotheses).

## Methods

### Ethical Considerations

The trial was approved by the University of New South Wales Human Research Ethics Committee (HC200105) and registered with the Australian New Zealand Clinical Trials Registry (ACTRN12620000489998p). Eligible participants were provided with a Participant Information Sheet and asked to provide consent using an online consent form. Participants received a 20 AUD (US$ 13.05) gift voucher for each survey completed (baseline, posttraining, and 3-month follow-up). Participant data ere deidentified.

### Design

A noninferiority randomized controlled trial was conducted to compare the R&R program with the QPR online program. Study outcomes were collected via self-report surveys at baseline, 2 weeks postbaseline, and at the 3-month follow-up. The study took place between July 2020 and March 2021 and was conducted online, with no face-to-face contact with participants.

### Participants and Sample Size

Eligible participants were required to be aged 18 years or over, have sufficient English literacy skills, and access to a computer, tablet, or smart phone to complete the training and surveys. To test that the R&R program was not inferior to the QPR program, a minimum of 170 participants in each group were needed to have 90% power to detect that the lower limit of a one-sided 97.5% CI (or equivalently a 95% two-sided CI) would be above the noninferiority limit of −5. A total attrition rate of 55% was expected at postintervention and follow-up, bringing the initial target sample size to 378 participants in total.

### Recruitment and Consent

Participants were recruited via two methods: a paid social media campaign of study advertisements on Facebook and Instagram, and the distribution of study details through the University of New South Wales (UNSW) and community organizations using flyers, posters, and websites. Advertisements contained a brief description of the training program and the study, and the inclusion criteria. Prospective participants who clicked on study advertisements were redirected to the study website where they were able to complete the eligibility screener.

### Procedure

All consenting participants were randomly allocated to the intervention or active comparator group using block randomization (4 per block). Participants were not blinded to their group allocation. Participants were given 1 week to complete the pretraining survey. If they did not complete it within this time, they were automatically withdrawn from the study. Participants had 2 weeks access to complete their assigned training. Automated invitations to complete the posttraining and follow-up surveys were sent at 2 weeks and 3 months after completing baseline, respectively. During this time, participants had 2 weeks to complete each survey, after which surveys were closed. Participants were encouraged to complete the surveys prior to closure via email reminders. For the baseline survey, email reminders were sent 3 days after the initial invitation. For the posttraining and follow-up surveys, reminders were sent 1 week after the initial survey invitations.

### Intervention Condition

Participants in the intervention condition received the R&R gatekeeper training program, described in the Introduction. R&R is an Australian program that teaches nonclinical gatekeepers to recognize and respond helpfully to someone exhibiting suicide warning signs and behaviors. It comprises four interactive learning modules, case studies, referral options, and a short test on the content covered in the training. The program is self-directed and takes 60 to 90 minutes to complete.

### Active Comparator Condition

Participants in the active comparator group received the QPR online gatekeeper training program [24]. The QPR online program is a mental health intervention that teaches lay and professional gatekeepers to recognize and respond helpfully to someone exhibiting suicide warning signs and behaviors. It is a US-based course that comprises an introductory video, presentations on QPR concepts and skills, and a short test on the content covered in the training. The online version of QPR is self-paced and users were encouraged to complete the program in their own time. On average, the program takes 60‐90 minutes to complete.

### Outcome Measures

#### Confidence

The primary outcome for this study was participants’ self-confidence in gatekeeper skills. A 6-item self-report questionnaire was created based on existing literature and the Mental Health First Aid (MHFA) guidelines for suicidal thoughts and behaviors to assess confidence. Items included statements such as, “I am able to recognize the signs that someone may be having thoughts of suicide,” and “I can work with someone to keep them safe from suicide” and were rated on a 5-point Likert scale ranging from “strongly disagree” (1) to “strongly agree” (5). Items were summed to give a total score ranging from 6 to 30. Higher scores indicated greater levels of confidence. In this study, the Cronbach α was 0.83, 0.92, and 0.87 at baseline, posttest, and follow-up, respectively.

#### Attitudes Towards Suicide Prevention

The ATSP (Attitudes Towards Suicide Prevention) scale is a 13-item self-report questionnaire used to assess health professionals’ attitudes towards suicide prevention. Items included statements such as “Suicide prevention is not my responsibility,” and “Since unemployment and poverty are the main causes of suicide, there is little that an individual can do to prevent it.” For the present study, 3 items not applicable to community members were removed (eg, “working with suicidal patients is rewarding”). The remaining 10 items were scored on a 5-point Likert scale ranging from “strongly disagree” (1) to “strongly agree” (5). Higher scores indicate more negative attitudes. In this study, the Cronbach α was 0.63, 0.73, and 0.72 at baseline, posttest, and follow-up, respectively.

#### Suicide Intervention Response

The SIRI-2 (Suicide Intervention Response Inventory revised) was designed to assess health professionals’ knowledge of appropriate responses to suicide. For the current study, participants were asked to evaluate the degree of appropriateness of two different response remarks to 5 suicidal item-statements, on a 7-point Likert scale ranging from +3 (highly appropriate response) through 0 (neither appropriate nor inappropriate response) to −3 (highly inappropriate response). As this is an edumetric scale, the Cronbach alpha is not reported.

#### Demographics

At baseline, participants reported how they had found out about the study (open text), their age (in years), gender identity (male, female, other, or prefer not to answer)*,* personal experience of suicide (Please select all statements that apply to you: I have had or continue to have suicidal thoughts, I have attempted suicide, I have cared for someone or continue to care for someone who is suicidal or who has attempted suicide, or I am bereaved by suicide) and whether they have received any prior suicide prevention training (No or Yes).

### Data Collection and Analysis

The BDI research engine was used to securely store collected data. Data were then exported to Microsoft Excel and SPSS (version 28.0.1.0; IBM Corp) for cleaning and analysis. Analyses were undertaken on an intent-to-treat basis, including all participants as randomized, regardless of whether participants completed the program or post and follow-up assessments. Descriptive statistics were used to summarize participant demographics and baseline scores for primary outcome of confidence, and secondary outcomes of attitudes and knowledge. For the primary hypothesis, a mixed-model repeated measures analyses of variance was conducted, using one-sided statistical tests due to the noninferiority design. This mixed-effects model provides an intention-to-treat analysis that accounts for all available data under the missing at random assumption [30]. A priori planned comparison of change from baseline to the posttraining endpoint (2 weeks) and follow-up (3 months) was used to test the primary hypothesis. The noninferiority margin for the primary outcome (confidence) was predefined as −5 points, and the confidence interval approach was used to assess noninferiority. This margin was selected using a heuristic approach, as the confidence scale had not been previously validated for noninferiority testing. Specifically, the margin was based on the assumption that a difference of less than one response choice per item would not be practically meaningful. An alpha level of 0.05 was used. For secondary hypotheses, the same mixed-effects models were used, but with two-sided statistical tests. In addition, we explored baseline factors associated with completion of the posttest and follow-up assessment using a logistic regression. All primary and secondary outcome variables assessed at pretest were included as independent variables in this regression analysis, along with age, gender, personal experience of suicide, and prior suicide prevention training history.

## Results

### Participants and Baseline Demographics

**Figure 3.**
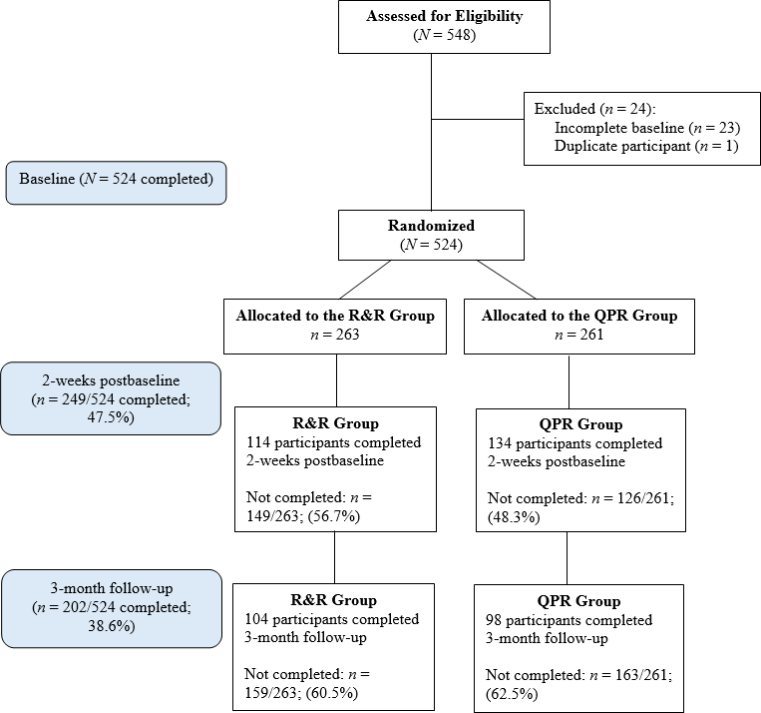
CONSORT study flow diagram of participant recruitment and participation. Note: Completed = participants who answered all questions in the assessment.

A total of 584 community members and UNSW staff self-registered for the study. From this, 524 participants completed baseline and were randomized. Two-hundred and thirty-six participants were assigned to the intervention condition and 261 were assigned to the active comparator condition (Figure 3, Checklist 1).

The majority of participants were female (446/524, 85.1%) with a mean (SD) age of 38.94 (13.12) years (range 18‐78 years; Table 1). At baseline, 42.7% of participants (224/524) reported that they have had or continue to have suicidal thoughts and 41.6% had experience caring for someone with suicidal ideation or attempt (218/524; Table 2). A small proportion (76/524, 14.5%) reported having previously attempted suicide. A quarter of participants reported having been bereaved by suicide (132/524, 25.2%). Over a quarter of participants reported that they had received prior suicide prevention training (152/524, 29%).

**Table 1.** Characteristics of the baseline sample.

Outcome	Total (N=524)				R&R group (n=263)				QPR group (n=261)			
	Range	Mean (SD)			Range	Mean (SD)			Range	Mean (SD)		
Age, years	18‐78	38.94 (13.12)			18‐75	39.00 (13.10)			18‐78	38.88 (13.17)		
Confidence score	6‐30	20.47 (4.24)			6‐30	20.22 (4.48)			6‐30	20.72 (3.98)		
Attitude towards suicide prevention score	11‐43	20.21 (4.35)			12‐43	20.46 (4.63)			11‐31	19.96 (4.04)		
Knowledge score	0‐10	7.81 (1.65)			1‐10	7.82 (1.62)			0‐10	7.80 (1.67)		

**Table 2.** Suicide experience and sex of participants at baseline.

Characteristics	Total (N=524), n (%)	R&R group (n=263), n (%)	QPR group (n=261), n (%)
Female	446 (85.1)	225 (85.6)	221 (84.7)
Have had or continue to have suicidal thoughts	224 (42.7)	108 (41.1)	116 (44.4)
Have attempted suicide	76 (14.5)	41 (15.6)	35 (13.4)
Have had or continue to care for someone who is suicidal or has attempted	218 (41.6)	106 (40.3)	112 (42.9)
Bereaved by suicide	132 (25.2)	63 (24)	69 (26.4)
Have received any prior suicide prevention training	152 (29)	75 (28.5)	77 (29.5)

### Baseline Factors Associated With Completion

Two baseline variables were significantly associated with study retention. Age was negatively associated with dropout (B=−0.016, SE=0.008, *P*=.035, OR=0.984) indicating that older participants were slightly more likely to complete all timepoints. Participants who have had or continue to care for someone who is suicidal or has attempted suicide were significantly more likely to complete the study (B=0.609, SE=0.254, *P*=.016, OR=1.838). Overall, the logistic regression model was not a strong predictor of study retention (*χ²*_13_=20.32, *P*=.088, *R²*=.054), explaining 5.4% of the variance in completion status.

### Primary and Secondary Outcomes

Baseline scores on confidence, attitudes, and knowledge did not differ significantly between groups (Table 3). There was a significant main effect of time for all outcomes such that both groups showed improvements in confidence, attitude, and knowledge scores between baseline and posttest (*P*<.001), and baseline and follow-up (*P*<.001). There were no significant changes in confidence, attitude, or knowledge scores between posttest and follow-up in either group.

**Table 3.** Estimated marginal means and standard errors of the confidence, attitudes towards suicide prevention, and knowledge measures.

Measure	Baseline		Posttraining		Follow-up	
	R&R group, mean (SE)	QPR group, mean (SE)	R&R group, mean (SE)	QPR group, mean (SE)	R&R group, mean (SE)	QPR group, mean (SE)
Confidence (primary outcome)	20.22 (0.25)	20.72 (0.25)	24.59 (0.36)	25.06 (0.34)	24.90 (0.40)	25.80 (0.40)
ATSP^a^	20.46 (0.27)	19.96 (0.27)	19.17^b^ (0.34)	16.68^b^ (0.33)	18.86^b^ (0.36)	16.91^b^ (0.37)
Knowledge	7.82 (0.10)	7.80 (0.10)	8.47 (0.13)	8.10 (0.12)	8.55 (0.14)	8.34 (0.14)

a ATSP=Attitudes towards suicide prevention.

b *P*<.05

The estimated raw mean differences (95% CI) in confidence scores between R&R and QPR were −0.46 (95% CI −1.44 to 0.51) at post-test, and −0.90 (95% CI −2.00 to 0.21) at follow-up. The lower limit of the 95% CI was above the prespecified noninferiority margin of −5 points at both timepoints, indicating that improvements in confidence in R&R at posttest and follow-up were noninferior to QPR.

No significant between-group differences were observed for knowledge scores at any timepoint. However, there was a significant between-group difference in attitude scores at posttest and follow-up (*ps*<.001), with participants in the QPR group showing greater improvement relative to the R&R group (between group Cohen *d*=0.46 at posttest; *d*=0.33 at follow-up).

## Discussion

### Principal Findings and Comparison With Previous Works

This noninferiority trial aimed to evaluate the efficacy of the R&R gatekeeper training program for improving confidence in gatekeeper skills, attitudes towards suicide prevention, and knowledge of appropriate responses to signs of suicide compared with the established QPR online program. The R&R gatekeeper training program is the first gatekeeper program that has been developed and evaluated to suit the Australian context. The results were consistent with the primary hypothesis that the R&R program was noninferior to QPR for self-reported confidence. Participants who received the R&R program showed improvements in their scores on confidence, attitudes, and knowledge at the end of the 2 weeks, with improvements in these scores present at the 3-month follow-up. Individuals who received the R&R gatekeeper training program showed similar improvements in their confidence and knowledge to those that received the QPR training program. Both groups improved on the measure of attitudes towards suicide, with the QPR group improving more at posttest and follow-up relative to baseline.

Both the R&R and QPR programs were found to have a positive effect on outcomes. Consistent with current literature, these findings suggest that e-learning gatekeeper training programs can improve users’ short- and medium-term confidence in identifying and supporting an individual experiencing thoughts of suicide, attitudes towards suicide prevention, and knowledge of appropriate responses to signs of suicide [23,31,32]. Further, the Australian R&R program was found to be noninferior to the established international QPR program for confidence. These findings imply that the addition of contextually relevant case studies and resources did not lessen the effectiveness of gatekeeper training in improving users’ confidence to recognize and respond to the signs of suicide compared to QPR.

While both groups showed improvements in attitudes towards suicide prevention, the magnitude of this improvement at posttest and follow-up was greater for the QPR group relative to the R&R group. Although this finding requires further investigation, it may reflect differences in content or interactivity. It is possible that a greater emphasis on connection with community and the meaningful impact of gatekeeper intervention may be necessary to provide similar change in attitudes such as felt responsibility and realistic achievability of suicide prevention. However, the small-moderate benefit of QPR on attitudinal change did not lead to differential increases in confidence or knowledge.

Future research should focus on assessing the long-term impacts (a 6-month follow-up or later) of the R&R program, given that the present study only assessed short- and medium-term impacts. Lancaster et al [23] found that, while showing initial improvements in knowledge, self-efficacy, and behavioral intention, participants showed a decrease in these outcomes between posttest and 6 months after completing the QPR training. As such, it is worth examining the long-term impacts of R&R compared to other established gatekeeper programs. Future research could examine whether participants accessed the PDF resources after training, and if indefinite access to the training content is sufficient to mitigate reductions in the effectiveness of training over time [11]. To further explore the efficacy of the R&R program in the Australian community, future studies should also assess changes in participants’ behavioral intention and gatekeeper behavior, preferably within a randomized controlled trial. This would involve measuring participants’ willingness to intervene, and their ability to recognize the signs of suicide, ask an individual if they are having thoughts of suicide, and the use of referral pathways. Examining behavioral outcomes is essential for progressing the broader research of gatekeeper training, given that supportive behaviors are rarely assessed despite being a necessary outcome in the success of any gatekeeper program [11]. Finally, it would be valuable to examine whether the inclusion of local referrals in the R&R program led to greater use of referral pathways compared to a gatekeeper program without this tailoring.

### Limitations

This study was impacted by greater attrition for survey completions than initially anticipated, with a 61.5% attrition rate at postintervention and a 69% attrition rate at follow-up. However, these rates were not significantly different between the groups. Logistic regression analyses indicated that younger participants were more likely to not complete all timepoints, but this effect was small. Future studies may benefit from increasing reimbursements for the completion of assessments or by incorporating additional survey reminders to minimize the attrition rate. The generalizability of the study’s findings is limited by the demographic composition of the sample, which consisted of 85.1% women. Further, the present study did not collect detailed demographic information such as the ethnicity, migrant status, or rurality of participants. As a result, the effectiveness of gatekeeper training may not be fully applicable to men or other gender diverse groups, and efficacy across other demographic factors is unknown. Diverse participants may have entered the trial with varied expectancies or goals about the intervention and its potential benefits that may have influenced how and why they chose to engage with the program, with the potential for differential impacts on efficacy. Future studies should consider assessing more nuanced demographic information and the expectancies and goals of participants enrolling in the study to allow for the evaluation of these possibilities. There may be some value in evaluating interventions with more well-defined and representative groups of suicide prevention stakeholders to allow for the elucidation of these issues and broader applicability of the study findings. Although the consideration of the diversity of potential users is difficult in a population-based trial [33], this may be critical when delivering gatekeeper training within a closed environment such as a healthcare setting or school district.

Additionally, our data analysis did not examine the relationship between training program completion and outcome measures on confidence, attitudes, and knowledge. It is worth assessing how program engagement influenced outcome measures. Further, the internal consistency of the attitude measure was moderate, suggesting the need for further development of suitable measures of gatekeeper training outcomes.

### Conclusions

This study was the first to rigorously evaluate an e-learning gatekeeper training program specific to the Australian context. Both programs were efficacious in improving gatekeepers’ confidence, attitudes, and knowledge. The R&R online program provides equivalent outcomes to the established QPR online program using collaboratively developed content suited to the Australian context.

## Supplementary material

10.2196/63204 Checklist 1 CONSORT (Consolidated Standards of Reporting Trials) checklist.

## Abbreviations

ATSP: Attitudes Towards Suicide Prevention
BDI: Black Dog Institute
LGBTQIA+: lesbian, gay, bisexual, transgender, queer/questioning, intersex, asexual
MHFA: Mental Health First Aid
QPR: Question, Persuade, Refer
R&R: Recognise and Respond
SIRI: Suicide Intervention Response Inventory
UNSW: University of New South Wales

## References

[1] World Health Organization (2023). Suicide.

[2] Australian Bureau of Statistics (2022). Causes of Death, Australia.

[3] Corrigan P (2004). How stigma interferes with mental health care. Am Psychol.

[4] Tang S, Reily NM, Arena AF (2021). People who die by suicide without receiving mental health services: a systematic review. Front Public Health.

[5] Stene-Larsen K, Reneflot A (2019). Contact with primary and mental health care prior to suicide: a systematic review of the literature from 2000 to 2017. Scand J Public Health.

[6] Joiner TE (2005). Why People Die by Suicide.

[7] Michelmore L, Hindley P (2012). Help-seeking for suicidal thoughts and self-harm in young people: a systematic review. Suicide Life Threat Behav.

[8] Krysinska K, Batterham PJ, Tye M (2016). Best strategies for reducing the suicide rate in Australia. Aust N Z J Psychiatry.

[9] Mann JJ, Apter A, Bertolote J (2005). Suicide prevention strategies: a systematic review. JAMA.

[10] World Health Organization (2014). Preventing suicide: a global imperative. https://www.who.int/publications/i/item/9789241564779.

[11] Holmes G, Clacy A, Hermens DF, Lagopoulos J (2021). The long-term efficacy of suicide prevention gatekeeper training: a systematic review. Arch Suicide Res.

[12] Burnette C, Ramchand R, Ayer L (2015). Gatekeeper training for suicide prevention. Rand Health Q.

[13] Torok M, Calear AL, Smart A, Nicolopoulos A, Wong Q (2019). Preventing adolescent suicide: a systematic review of the effectiveness and change mechanisms of suicide prevention gatekeeping training programs for teachers and parents. J Adolesc.

[14] QPR Institute (2017). What is QPR? QPR Institute.

[15] Reis C, Cornell D (2008). An evaluation of suicide gatekeeper training for school counselors and teachers. Professional School Counseling.

[16] Cross W, Matthieu MM, Cerel J, Knox KL (2007). Proximate outcomes of gatekeeper training for suicide prevention in the workplace. Suicide Life Threat Behav.

[17] Cross W, Matthieu MM, Lezine D, Knox KL (2010). Does a brief suicide prevention gatekeeper training program enhance observed skills?. Crisis.

[18] Matthieu MM, Cross W, Batres AR, Flora CM, Knox KL (2008). Evaluation of gatekeeper training for suicide prevention in veterans. Arch Suicide Res.

[19] Aldrich RS, Wilde J, Miller E (2018). The effectiveness of QPR suicide prevention training. Health Educ J.

[20] Litteken C, Sale E (2018). Long-term effectiveness of the question, persuade, refer (QPR) suicide prevention gatekeeper training program: lessons from Missouri. Community Ment Health J.

[21] Quinnett P (2007). QPR gatekeeper training for suicide prevention: the model, rationale, and theory. https://qprinstitute.com/uploads/main/QPR-Theory-Paper-Master-Final-2019.pdf.

[22] Wyman PA, Brown CH, Inman J (2008). Randomized trial of a gatekeeper program for suicide prevention: 1-year impact on secondary school staff. J Consult Clin Psychol.

[23] Lancaster PG, Moore JT, Putter SE (2014). Feasibility of a web-based gatekeeper training: implications for suicide prevention. Suicide Life Threat Behav.

[24] Shand F, Torok M, Cockayne N (2020). Protocol for a stepped-wedge, cluster randomized controlled trial of the LifeSpan suicide prevention trial in four communities in New South Wales, Australia. Trials.

[25] Zaheer J, Eynan R, Lam JSH, Grundland M, Links PS (2018). “We went out to explore, but gained nothing but illness”: immigration expectations, reality, risk and resilience in Chinese-Canadian women with a history of suicide-related behaviour. Cult Med Psychiatry.

[26] Bowden M, McCoy A, Reavley N (2020). Suicidality and suicide prevention in culturally and linguistically diverse (CALD) communities: a systematic review. Int J Ment Health.

[27] Suicide Prevention Australia (2022). State of the nation in suicide prevention: a survey of the suicide prevention sector. https://www.suicidepreventionaust.org/wp-content/uploads/2022/09/SPA_StateNationReport_2022_FINAL-2.pdf.

[28] Muñoz RF (2010). Using evidence-based internet interventions to reduce health disparities worldwide. J Med Internet Res.

[29] Baumann M, Stargardt T, Frey S (2020). Cost-utility of internet-based cognitive behavioral therapy in unipolar depression: a Markov model simulation. Appl Health Econ Health Policy.

[30] Siddiqui O, Hung HMJ, O’Neill R (2009). MMRM vs. LOCF: a comprehensive comparison based on simulation study and 25 NDA datasets. J Biopharm Stat.

[31] Ghoncheh R, Gould MS, Twisk JW, Kerkhof AJ, Koot HM (2016). Efficacy of adolescent suicide prevention e-learning modules for gatekeepers: a randomized controlled trial. JMIR Ment Health.

[32] Holmes G, Clacy A, Hamilton A, Kõlves K (2024). Online versus in-person gatekeeper suicide prevention training: comparison in a community sample. J Ment Health.

[33] Sit HF, Hall BJ, Li SX (2023). Cultural adaptation of interventions for common mental disorders. Lancet Psychiatry.

